# Closed-Form Pseudolinear Estimators for DRSS-AOA Localization

**DOI:** 10.3390/s21217159

**Published:** 2021-10-28

**Authors:** Jun Li, Kutluyil Dogancay, Hatem Hmam

**Affiliations:** 1 UniSA STEM, University of South Australia, Mawson Lakes Campus, Mawson Lakes, SA 5095, Australia; jun.li@mymail.unisa.edu.au; 2 Defence Science & Technology Group, Cyber and Electronic Warfare Division, Edinburgh, SA 5111, Australia; Hatem.Hmam@dst.defence.gov.au

**Keywords:** hybrid localization, differential received signal strength localization, bearings-only localization, maximum likelihood, pseudolinear estimator, least squares, instrumental variables

## Abstract

This paper investigates the hybrid source localization problem using differential received signal strength (DRSS) and angle of arrival (AOA) measurements. The main advantage of hybrid measurements is to improve the localization accuracy with respect to a single sensor modality. For sufficiently short wavelengths, AOA sensors can be constructed with size, weight, power and cost (SWAP-C) requirements in mind, making the proposed hybrid DRSS-AOA sensing feasible at a low cost. Firstly the maximum likelihood estimation solution is derived, which is computationally expensive and likely to become unstable for large noise levels. Then a novel closed-form pseudolinear estimation method is developed by incorporating the AOA measurements into a linearized form of DRSS equations. This method eliminates the nuisance parameter associated with linearized DRSS equations, hence improving the estimation performance. The estimation bias arising from the injection of measurement noise into the pseudolinear data matrix is examined. The method of instrumental variables is employed to reduce this bias. As the performance of the resulting weighted instrumental variable (WIV) estimator depends on the correlation between the IV matrix and data matrix, a selected-hybrid-measurement WIV (SHM-WIV) estimator is proposed to maintain a strong correlation. The superior bias and mean-squared error performance of the new SHM-WIV estimator is illustrated with simulation examples.

## 1. Introduction

Source localization plays an important role in wireless sensor networks, providing location information about sensor nodes and emitters from sensor measurements. Several sensor modalities have been considered for source localization such as angle of arrival (AOA), differential received signal strength (DRSS), time of arrival, time difference of arrival, and frequency difference of arrival. This paper develops new closed-form source localization methods using hybrid DRSS-AOA measurements, built on pseudolinear DRSS and AOA equations combined in a unique way to eliminate the undesirable nuisance parameter associated with pseudolinear DRSS equations.

Source localization and tracking using AOA measurements has been an active research area for several decades. The nonlinear relationship between source location and sensor measurements is the key challenge with AOA localization. This challenge is also shared to varying degrees by other sensor modalities. The pioneering work of Stansfield [1] established the basis for most AOA localization algorithms proposed to this day. The Stansfield estimator is a weighted least-squares estimator which requires prior knowledge of the source range from each AOA sensor. The maximum likelihood estimator (MLE) for AOA localization [2,3] solves a nonlinear optimization problem representing the log-likelihood function by using iterative algorithms such as the Gauss–Newton and Levenberg–Marquardt algorithms [4]. While the MLE enjoys asymptotic efficiency and unbiasedness, it is computationally expensive as a result of iterative computations and can suffer from divergence issues caused by poor initialization and threshold effect [5]. This makes the MLE unsuitable for most practical implementations.

The pseudolinear estimator (PLE) was developed as a closed-from alternative to the MLE, where the nonlinear estimation problem is converted into a linear problem, allowing for a computationally simple least squares solution [6]. An estimator identical to the PLE was also presented in [7]. Despite its simplicity, the PLE was discovered to produce biased estimates [3,8]. This led to an intensive research effort to reduce or eliminate the PLE bias (see, e.g., [9,10,11,12,13,14,15,16,17,18]). Among those, two ideas that have gained popularity are bias compensation and weighted instrumental variables (WIV) [13]. The bias compensation method is based on estimation and subtraction of bias, whereas the method of weighted instrumental variables reduces bias by introducing an instrumental variable matrix, which is statistically independent of measurement noise, into the WLS solution. In [19], a closed-form AOA localization algorithm is presented with no prior knowledge of AOA measurement variances. AOA-based self localization algorithms built on the PLE were developed in [20,21].

Received signal strength (RSS) localization offers a low-cost alternative to other localization systems as RSS measurements are readily available in most wireless systems. As different from RSS localization, DRSS localization methods use the differences between RSS measurements taken at pairs of sensor nodes, which eliminates the requirement for prior knowledge of transmit power at the source. This makes DRSS better suited for practical applications [22,23,24]. DRSS values, measured in dB, correspond to the ratio of source-sensor ranges from two sensors. Therefore, the DRSS source localization problem is reduced into a circular intersection problem where each circle represents a locus of possible source locations with the same range ratio from a pair of sensors as given by the corresponding DRSS measurement (the Apollonian circles theorem [25]). The research on DRSS localization has also focused on solving nonlinear and nonconvex optimization problems. Some of the existing solutions for DRSS localization include the MLE [22], weighted least-squares (WLS) [24,26,27], the generalized trust region subproblem (GTRS) estimator [24], semi-definite programming [24,28] and the PLE with bias reduction [29,30]. The derivation of DRSS equations and a summary of basic methods for DRSS localization are provided in [31].

Hybrid localization algorithms combining AOA and RSS measurements have been reported in the open literature. The work in [32,33,34,35,36,37] uses different linearization methods to convert both RSS and AOA equations into a linear form with a common unknown vector. The source location is easily obtained by using the WLS. However, the WLS estimates obtained from linearized RSS measurements have a bias problem, which has not been widely discussed in the current research. In contrast, for hybrid localization methods using TDOA-AOA measurements, besides the MLE and the WLS solution [38,39], the PLE with a bias reduction method has also been developed. For example, the work in [40] proposes bias compensation and weighted instrumental variable methods to reduce the bias.

Hybrid DRSS-AOA localization has not attracted much research despite the great potential it offers as a feasible and low-cost localization method compared with RSS-AOA and TDOA-AOA methods. The work in [41] proposes a hybrid RSS-AOA localization algorithm that treats the transmit power as unknown parameter, based on second-order cone programming relaxation techniques. In this paper, we present new hybrid localization algorithms using DRSS and AOA measurements based on the PLE and its instrumental variable variants. In DRSS localization, the knowledge of source transmit power is not required and therefore its estimation is not necessary. The conventional MLE is also derived, which is capable of achieving the Cramer–Rao lower bound (CRLB) with low bias, but has convergence problems and suffers from high computational complexity. The PLE is developed by converting the nonlinear measurement equations into linear form. The PLE is a closed-form estimator and has the advantage of low computational difficulty. In addition, the proposed PLE is free of nuisance parameter introduced into the linearized DRSS equations, thereby avoiding complications with constrained parameters in the solution vector. The PLE can be solved by using least squares (LS) and WLS. However, both these solutions have a bias problem due to the injection of measurement noise into the data matrix during the linearization process. The bias problem can be mitigated by introducing an instrumental variable matrix which correlates strongly with the data matrix and is independent of noise. However, when the measurement noise is high, the correlation between the instrumental variable matrix and data matrix is weakened. This can be remedied by adopting a selective measurement method when constructing the instrumental variable matrix, resulting in the selective-hybrid-measurement WIV (SHM-WIV) estimator. The SHM-WIV estimator is shown to outperform the MLE, LS, WLS and WIV estimators by way of simulation examples. The multipath effects on AOA and DRSS measurements are ignored even though shadowing effects are taken into account by lognormal noise on DRSS measurements.

The paper is organized as follows. Section 2 defines the hybrid localization problem addressed in this paper. The MLE and CRLB for the hybrid DRSS-AOA localization problem are presented in Section 3. In Section 4, linearized AOA and DRSS measurement equations are derived, and it is shown how the nuisance parameter present in the linearized DRSS measurement equation can be eliminated by incorporating the AOA measurements. The hybrid DRSS-AOA equation free of nuisance parameter is then solved using LS, WLS, WIV and SHM-WIV in Section 5. Comparative simulation results are demonstrated and discussed in Section 6. Concluding remarks are made in Section 7.

## 2. Problem Definition

**Figure 1:**
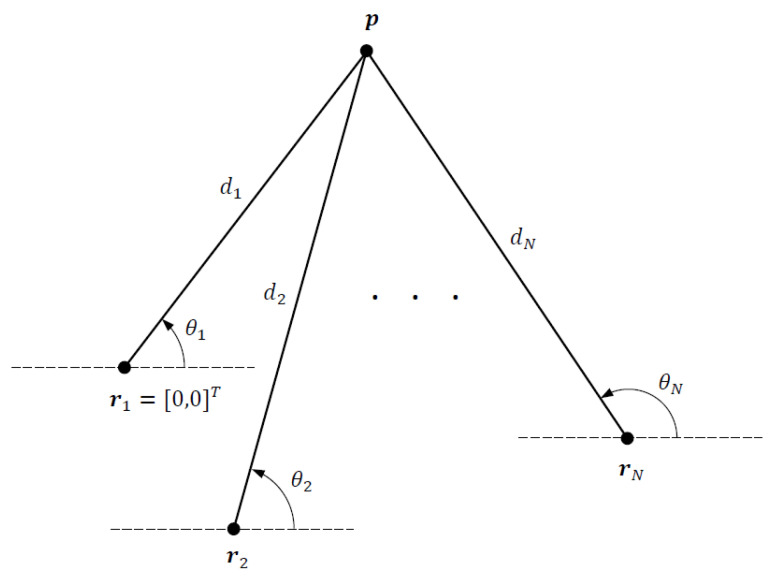
DRSS-AOA localization geometry.

We consider a 2D DRSS-AOA localization problem depicted in Figure 1, where the objective is to estimate the unknown source location $p={\left[x,y\right]}^{T}$ from DRSS and AOA measurements collected by *N* sensors at fixed and known locations $r_{i}={\left[x_{i},y_{i}\right]}^{T}$, i=1,…,N. The distance between the source and a sensor is given by $d_{i}=∥d_{i}∥$ where $d_{i}=p-r_{i}$ and ∥·∥ denotes the Euclidean norm. Letting $r_{1}$ be the reference sensor location for DRSS measurements, we set $r_{1}=0$ after appropriate geometric translation with no loss of generality.

The noisy AOA measurements at sensor *i* are given by

(1) $${\tilde{\theta }}_{i}=\theta_{i}+n_{i},\;i=1,\ldots ,N,$$

where $n_{i}∼N\left(0,\sigma_{\theta_{i}}^{2}\right)$ is an independent additive noise with zero mean and variance $\sigma_{\theta_{i}}^{2}$. The true angle $\theta_{i}$ is

(2) $$\theta_{i}=\tan^{-1}\left(y-y_{i},x-x_{i}\right),\;\theta_{i}\in \left(-\pi ,\pi \right]$$

where $\tan^{-1}$ is the 4-quadrant inverse tangent. The covariance matrix of the AOA measurements $\left[{\tilde{\theta }}_{1},\ldots ,{\tilde{\theta }}_{N}\right]$ is a diagonal matrix:

(3) $$W_{\mathrm{AOA}}=\mathrm{diag}\left(\sigma_{\theta_{1}}^{2},\;\ldots ,\;\sigma_{\theta_{N}}^{2}\right).$$

The power difference (DRSS) measurements with respect to the reference sensor at $r_{1}$ follow the propagation path loss model [24,26,29,30,42]

(4) $${\tilde{p}}_{i,1}=p_{i,1}+\epsilon_{i,1},\;i=2,\ldots ,N,$$

where $p_{i,1}=10\gamma \log_{10}\frac{d_{1}}{d_{i}}$ is the true power difference between sensor *i* and the reference sensor (in dBm or dBW), γ is the path loss exponent which is assumed known a priori, and $\epsilon_{i,1}∼N\left(0,\sigma_{p_{1}}^{2}+\sigma_{p_{i}}^{2}\right)$ is the log-normal noise representing shadowing effects with variance $\sigma_{p_{1}}^{2}+\sigma_{p_{i}}^{2}$, which is the sum of RSS log-normal noise variances at $r_{1}$ and $r_{i}$. The covariance matrix of the DRSS measurements $\left[{\tilde{p}}_{2,1},\ldots ,{\tilde{p}}_{N,1}\right]$ is

(5) $$W_{\mathrm{DRSS}}=\sigma_{p_{1}}^{2}1_{N-1}+\mathrm{diag}\left(\sigma_{p_{2}}^{2},\ldots ,\sigma_{p_{N}}^{2}\right)$$

where $1_{N-1}$ is an (N−1)×(N−1) matrix of ones.

The (2N−1)×1 hybrid measurement vector combining the AOA and DRRS measurements is

(6) $$\tilde{\psi }=\psi +\beta ,$$

where

(7a) $$\tilde{\psi }={\left[{\tilde{\theta }}_{1},\;\ldots ,\;{\tilde{\theta }}_{N},{\tilde{p}}_{2,1},\;\ldots ,\;{\tilde{p}}_{N,1}\right]}^{T},$$

(7b) $$\psi ={\left[\theta_{1},\;\ldots ,\;,\theta_{N},p_{2,1},\;\ldots ,\;p_{N,1}\right]}^{T},$$

(7c) $$\beta ={\left[n_{1},\;\ldots ,\;n_{N},\epsilon_{2,1},\;\ldots ,\;\epsilon_{N,1}\right]}^{T}.$$

The covariance matrix of β is a (2N−1)×(2N−1) block-diagonal matrix

(8) $$W=E\left\{\beta \beta^{T}\right\}=\left[\begin{array}{cc} W_{\mathrm{AOA}} & 0\\ 0 & W_{\mathrm{DRSS}} \end{array}\right].$$

Observe that the AOA and DRSS measurement errors are not correlated. This is because the AOA measurement errors arise from thermal noise and possibly some interference at sensors while the log-normal noise in DRSS measurements is caused by shadowing. The two noise sources are physically independent phenomena.

## 3. Maximum Likelihood Estimator

The likelihood function for the hybrid measurements is a multivariate Gaussian pdf [43], which is given by

(9) $$\begin{array}{cc} p(\tilde{\psi }|\hat{p}) & =\frac{1}{{\left(2\pi \right)}^{(2N-1)/2}{\left|W\right|}^{1/2}}\\ & \;\times \exp\left\{-\frac{1}{2}{\left(\tilde{\psi }-\psi \left(\hat{p}\right)\right)}^{T}W^{-1}\left(\tilde{\psi }-\psi \left(\hat{p}\right)\right)\right\}, \end{array}$$

where |·| denotes matrix determinant or scalar absolute value, and

(10) $$\psi \left(\hat{p}\right)={\left[\theta_{1}\left(\hat{p}\right),\;\ldots ,\;,\theta_{N}\left(\hat{p}\right),p_{2,1}\left(\hat{p}\right),\;\ldots ,\;p_{N,1}\left(\hat{p}\right)\right]}^{T}$$

is the (2N−1)×1 vector of DRSS-AOA estimates constructed by substituting the estimated source location $\hat{p}={\left[\hat{x},\hat{y}\right]}^{T}$ for the true source location p:

(11a) $$\theta_{i}\left(\hat{p}\right)=\tan^{-1}\left(\hat{y}-y_{i},\hat{x}-x_{i}\right),\;\theta_{i}\left(\hat{p}\right)\in \left(-\pi ,\pi \right],\;i=1,\ldots ,N,$$

(11b) $$p_{i,1}\left(\hat{p}\right)=-10\gamma \log_{10}\frac{∥\hat{p}-r_{i}∥}{∥\hat{p}∥},\;i=2,\ldots ,N.$$

The maximum likelihood estimate (MLE) of the source location is obtained by maximizing the log-likelihood function $\ln p(\tilde{\psi }|\hat{p})$ over $\hat{p}$, which is equivalent to

(12) $${\hat{p}}_{\mathrm{ML}}=\underset{p\in R^{2}}{arg min}h^{T}\left(p\right)W^{-1}h\left(p\right),$$

where

(13) $$h\left(p\right)=\tilde{\psi }-\psi \left(p\right).$$

The nonlinear minimization problem in (12) can be solved numerically by an iterative search algorithm such as the steepest-descent, Levenberg–Marquardt, trust region and Gauss–Newton method [44]. In this paper, the Gauss–Newton method is adopted, which calculates the MLE using the following iterations:

(14) $$\hat{p}\left(j+1\right)=\hat{p}\left(j\right)+{\left(J^{T}\left(j\right)W^{-1}J\left(j\right)\right)}^{-1}J^{T}\left(j\right)W^{-1}h\left(\hat{p}\left(j\right)\right),\;j=0,1,\ldots$$

Here J(j) is the (2N−1)×2 Jacobian matrix of $\psi (\hat{p})$ evaluated at $p=\hat{p}\left(j\right)$:

(15) $$J\left(j\right)={\left[J_{\theta_{1}}^{T}\left(j\right),\ldots ,J_{\theta_{N}}^{T}\left(j\right),J_{p_{2,1}}^{T}\left(j\right),\ldots ,J_{p_{N,1}}^{T}\left(j\right)\right]}^{T},$$

where

(16a) $$J_{\theta_{k}}\left(j\right)=\frac{\left[-\sin\theta_{i}\left(\hat{p}\left(j\right)\right),\cos\theta_{i}\left(\hat{p}\left(j\right)\right)\right]}{∥\hat{p}\left(j\right)-r_{i}∥},\;i=1,\ldots ,N$$

(16b) $$\begin{array}{cc} J_{p_{i,1}}\left(j\right) & =\frac{10\gamma }{\ln(10)}\left(\frac{{\left(r_{i}-\hat{p}\left(j\right)\right)}^{T}}{∥r_{i}-\hat{p}{\left(j\right)∥}_{2}^{2}}+\frac{{\hat{p}}^{T}\left(j\right)}{∥\hat{p}{\left(j\right)∥}_{2}^{2}}\right)\\ \; & =\frac{10\gamma }{\ln(10)}{\left[\begin{array}{c} -\frac{\cos\theta_{i}\left(\hat{p}\left(j\right)\right)}{∥\hat{p}\left(j\right)-r_{i}∥}+\frac{\cos\theta_{1}\left(\hat{p}\left(j\right)\right)}{∥\hat{p}\left(j\right)∥}\\ -\frac{\sin\theta_{i}\left(\hat{p}\left(j\right)\right)}{∥\hat{p}\left(j\right)-r_{i}∥}+\frac{\sin\theta_{1}\hat{p}\left(j\right)}{∥\hat{p}\left(j\right)∥} \end{array}\right]}^{T},\;i=2,3\ldots ,N. \end{array}$$

The GN is initialized by $\hat{p}\left(0\right)$, which needs to be selected carefully.

Being asymptotically efficient and unbiased, the MLE is often considered to be a benchmark in performance comparisons. However, the iterative methods used in MLE calculation can diverge if they are poorly initialized or the noise is too large, causing threshold effects (sharp degradation in estimation performance as the measurement noise increases above a threshold value). Furthermore, the MLE algorithms have a large computational complexity.

The Cramer–Rao lower bound for the hybrid DRSS-AOA localization problem is given by [43]

(17) $$\mathrm{CRLB}={\left(J_{0}^{T}W^{-1}J_{0}\right)}^{-1},$$

where $J_{0}$ is the Jacobian matrix in (15) evaluated at the true source location p.

## 4. Pseudolinear Equations for Hybrid Measurements

### 4.1. Linearized AOA Equations

According to [6,40], the pseudolinear form for AOA measurements is

(18) $$A_{\theta_{i}}p=b_{\theta_{i}}+e_{\theta_{i}},\;i=1,\ldots ,N$$

where

(19a) $$A_{\theta_{i}}=\left[\sin\;{\tilde{\theta }}_{i},-\cos\;{\tilde{\theta }}_{i}\right],$$

(19b) $$b_{\theta_{i}}=\left[\sin\;{\tilde{\theta }}_{i},-\cos\;{\tilde{\theta }}_{i}\right]r_{i},$$

(19c) $$e_{\theta_{i}}=d_{i}\sin\;n_{i}\approx d_{i}n_{i}.$$

The approximation in (19c) is valid for sufficiently small AOA measurement noise.

### 4.2. Linearized DRSS Equations

The DRSS measurement Equation (4) can be rewritten as

(20) $$10^{\frac{{\tilde{p}}_{i,1}}{10\gamma }}d_{i}=10^{\frac{\epsilon_{i,1}}{10\gamma }}d_{1},\;i=2,\ldots ,N.$$

Squaring both sides of the above equation yields

(21) $$A_{i,1}y=b_{i}+e_{i},\;i=2,\ldots ,N$$

where

(22a) $$\begin{array}{cc} A_{i,1} & =[-2\times 10^{\frac{{\tilde{p}}_{i,1}}{5\gamma }}r_{i}^{T},\;10^{\frac{{\tilde{p}}_{i,1}}{5\gamma }}-1] \end{array}$$

(22b) $$\begin{array}{cc} y & =\left[\begin{array}{c} p\\ {∥p∥}^{2} \end{array}\right] \end{array}$$

(22c) $$\begin{array}{cc} b_{i} & =-10^{\frac{{\tilde{p}}_{i,1}}{5\gamma }}{∥r_{i}∥}^{2} \end{array}$$

(22d) $$\begin{array}{cc} e_{i} & =\left(10^{\frac{\epsilon_{i,1}}{5\gamma }}-1\right)d_{1}^{2} \end{array}$$

A key challenge with the linearized DRSS Equation (21) is the presence of a nuisance parameter, viz., ${∥p∥}^{2}$, in y, that depends on the source location, thereby creating an undesirable nonlinear constraint in the solution. This constraint must be imposed on the estimate of y to assure good estimation performance.

### 4.3. Linearized DRSS-AOA Equations

Here we show how the nuisance parameter in the linearized DRSS equation can be eliminated by using hybrid DRSS-AOA measurements, leading to a linear matrix equation free of nuisance parameter and nonlinear constraints. To do this, first consider the noiseless DRSS equation

(23) $$p_{i,1}=-10\gamma \log_{10}\frac{d_{i}}{d_{1}},\;i=2,\ldots ,N$$

which can be rewritten as

(24) $$10^{-\frac{p_{i,1}}{10\gamma }}∥p∥=d_{i}.$$

**Figure 2:**
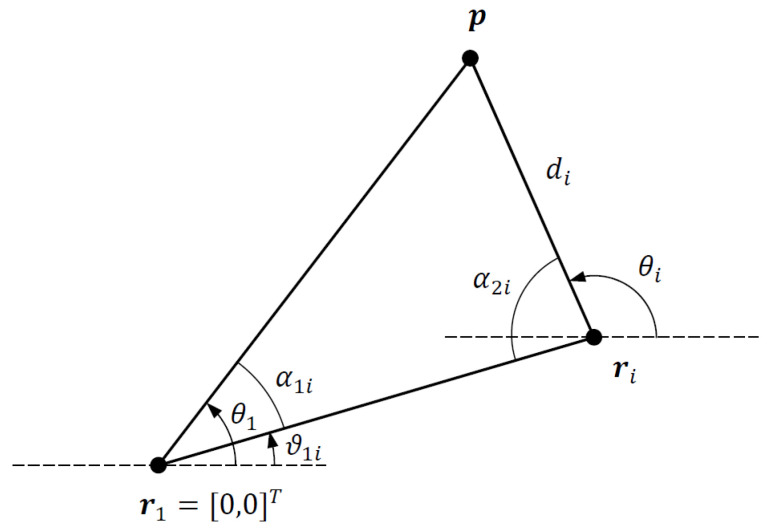
Triangle formed by corner points p, $r_{1}$ and $r_{i}$.

Next, consider the triangle formed by the corner points p, $r_{1}$ and $r_{i}$, which shown in Figure 2. From the dot products $r_{i}\cdot p$ and $r_{i}\cdot d_{i}$, we obtain

(25a) $$\cos\alpha_{1i}=\frac{r_{i}^{T}p}{∥r_{i}∥∥p∥}\Rightarrow ∥p∥=\frac{r_{i}^{T}p}{∥r_{i}∥\cos\alpha_{1i}},$$

(25b) $$\cos\alpha_{2i}=-\frac{r_{i}^{T}d_{i}}{∥r_{i}∥d_{i}}\Rightarrow d_{i}=-\frac{r_{i}^{T}d_{i}}{∥r_{i}∥\cos\alpha_{2i}}.$$

The angles of the triangle $\alpha_{1i}$ and $\alpha_{2i}$ are easily obtained from the AOA angles $\theta_{1}$ and $\theta_{i}$ as follows:

(26a) $$\begin{array}{cc} \vartheta_{1i} & =∠r_{i} \end{array}$$

(26b) $$\begin{array}{cc} \alpha_{1i} & =\theta_{1}-\vartheta_{1i},\;-\pi <\alpha_{1i}\leq \pi \end{array}$$

(26c) $$\begin{array}{cc} \alpha_{2i} & =\pi -\theta_{i}+\vartheta_{1i},\;-\pi <\alpha_{2i}\leq \pi \end{array}$$

where *∠* denotes the vector angle. Note that both $\alpha_{1i}$ and $\alpha_{2i}$ are wrapped to the interval (−π,π].

Substituting (25a) and (25b) into (24) yields

(27a) $$\begin{array}{c} 10^{-\frac{p_{i,1}}{10\gamma }}\frac{r_{i}^{T}p}{∥r_{i}∥\cos\alpha_{1i}}=-\frac{r_{i}^{T}d_{i}}{∥r_{i}∥\cos\alpha_{2i}} \end{array}$$

(27b) $$\begin{array}{c} 10^{-\frac{p_{i,1}}{10\gamma }}r_{i}^{T}p\cos\alpha_{2i}=-r_{i}^{T}d_{i}\cos\alpha_{1i}. \end{array}$$

Finally, plugging $d_{i}=p-r_{i}$ into (27b), we obtain the following linearized DRSS equation incorporating AOA, which is free of nuisance parameter:

(28) $${\bar{A}}_{p_{i,1}}p={\bar{b}}_{p_{i,1}}$$

where

$${\bar{A}}_{p_{i,1}}=\left(10^{-\frac{p_{i,1}}{10\gamma }}\cos\alpha_{2i}+\cos\alpha_{1i}\right)r_{i}^{T}$$

and

$${\bar{b}}_{p_{i,1}}={∥r_{i}∥}^{2}\cos\alpha_{1i}.$$

Replacing the true AOA and DRSS values with noisy measurements, (28) becomes

(29) $$A_{p_{i,1}}p=b_{p_{i,1}}+e_{p_{i,1}},$$

where $e_{p_{i,1}}$ is given by (A5) in Appendix A, and

(30a) $$\begin{array}{cc} A_{p_{i,1}} & =\left(10^{-\frac{{\tilde{p}}_{i,1}}{10\gamma }}\cos{\tilde{\alpha }}_{2i}+\cos{\tilde{\alpha }}_{1i}\right)r_{i}^{T} \end{array}$$

(30b) $$\begin{array}{cc} b_{p_{i,1}} & =∥r_{i}{∥}^{2}\cos{\tilde{\alpha }}_{1i} \end{array}$$

(30c) $$\begin{array}{cc} {\tilde{\alpha }}_{1i} & =\alpha_{1i}+n_{1} \end{array}$$

(30d) $$\begin{array}{cc} {\tilde{\alpha }}_{2i} & =\alpha_{2i}-n_{i}. \end{array}$$

As AOA and DRSS measurement errors are zero mean, we have

(31) $$E\{e_{p_{i,k}}\}\approx 0.$$

Stacking *N* AOA measurements and N−1 DRSS measurements, we obtain the linearized DRSS-AOA matrix equation:

(32) Ap=b+e,

where

(33a) $$\begin{array}{cc} A & ={\left[A_{\theta_{1}}^{T},\cdots ,A_{\theta_{N}}^{T},A_{p_{2,1}}^{T},\cdots ,A_{p_{N,1}}^{T}\right]}^{T}, \end{array}$$

(33b) $$\begin{array}{cc} b & ={\left[b_{\theta_{1}},\cdots ,b_{\theta_{N}},b_{p_{2,1}},\cdots ,b_{p_{N,1}}\right]}^{T}, \end{array}$$

(33c) $$\begin{array}{cc} e & ={\left[e_{\theta_{1}},\cdots ,e_{\theta_{N}},e_{p_{2,1}},\cdots ,e_{p_{N,1}}\right]}^{T} \end{array}$$

and

(34) E{e}≈0.

Note that (32) does not have a nuisance parameter. Therefore it can be solved without any constraint on the unknown vector as described in the next section.

## 5. Hybrid Pseudolinear Estimators

### 5.1. LS Solution and Bias Analysis

The least-squares solution for the linear matrix equation Ap≈b (see (32)) is [43]

(35a) $$\begin{array}{cc} {\hat{p}}_{\mathrm{LS}} & =\underset{p\in R^{2}}{arg min}{∥Ap-b∥}^{2} \end{array}$$

(35b) $$\begin{array}{cc} \; & ={\left(A^{T}A\right)}^{-1}A^{T}b. \end{array}$$

Substituting (32) into (35b), the least-squares estimate in terms of the pseudolinear noise vector e can be written as

(36) $$\begin{array}{cc} {\hat{p}}_{\mathrm{LS}} & ={\left(A^{T}A\right)}^{-1}A^{T}b\\ \; & ={\left(A^{T}A\right)}^{-1}A^{T}\left(Ap-e\right)\\ \; & =p-{\left(A^{T}A\right)}^{-1}A^{T}e. \end{array}$$

The least-squares estimation bias is

(37) $$\delta_{\mathrm{LS}}=E\left\{{\hat{p}}_{\mathrm{LS}}\right\}-p=-E\left\{{\left(A^{T}A\right)}^{-1}A^{T}e\right\},$$

and the error covariance matrix of the estimate is

(38) $$\begin{array}{cc} C_{\mathrm{LS}} & =E\{\left({\hat{p}}_{\mathrm{LS}}-p\right){\left({\hat{p}}_{\mathrm{LS}}-p\right)}^{T}\}\\ \; & =E\{{\left(A^{T}A\right)}^{-1}A^{T}ee^{T}A{\left(A^{T}A\right)}^{-1}\}. \end{array}$$

For sufficiently large *N* and under mild assumptions, Slutsky’s theorem [45] allows (37) to be approximated by the product of expectations:

(39) $$\delta_{\mathrm{LS}}\approx -E{\left\{\frac{A^{T}A}{2N-1}\right\}}^{-1}E\left\{\frac{A^{T}e}{2N-1}\right\}.$$

Using (33), the cross-correlation between A and e is

(40) $$E\left\{A^{T}e\right\}=\sum_{i=1}^{N}E\left\{A_{\theta_{i}}^{T}e_{\theta_{i}}\right\}+\sum_{i=2}^{N}E\left\{A_{p_{i,1}}^{T}e_{p_{i,1}}\right\}.$$

According to (19a) and (19c), even for small AOA noise, we have [40]

(41) $$E\left\{A_{\theta_{i}}^{T}e_{\theta_{i}}\right\}\approx \sigma_{\theta_{i}}^{2}d_{i}\neq 0.$$

An approximate expression for $E\{A_{p_{i,1}}^{T}e_{p_{i,1}}\}$ can be derived from (30a) and (A4). Firstly, expanding the cosine terms of $A_{p_{i,1}}$ and approximating $A_{p_{i,1}}$ using (A3), we obtain

(42) $$\begin{array}{cc} A_{p_{i,1}} & \approx (C_{1i}\cos\alpha_{2i}+\cos\alpha_{1i}-C_{2i}\epsilon_{i,1}\cos\alpha_{2i}\\ \; & \;+C_{1i}n_{i}\sin\alpha_{2i}-n_{1}\sin\alpha_{1i}\\ \; & \;-C_{1i}\frac{n_{i}^{2}}{2}\cos\alpha_{2i}+C_{3i}\epsilon_{i,1}^{2}\cos\alpha_{2i}\\ \; & \;-C_{2i}n_{i}\epsilon_{i,1}\sin\alpha_{2i}-\frac{n_{1}^{2}}{2}\cos\alpha_{1i}\\ \; & \;+C_{2i}\frac{n_{i}^{2}}{2}\epsilon_{i,1}\cos\alpha_{2i}+C_{3i}n_{i}\epsilon_{i,1}^{2}\sin\alpha_{2i}\\ \; & \;-C_{3i}\frac{n_{i}^{2}}{2}\epsilon_{i,1}^{2}\cos\alpha_{2i})r_{i}^{T}. \end{array}$$

Taking the expectation of $A_{p_{i,1}}^{T}e_{p_{i,1}}$ yields

(43) $$\begin{array}{cc} E\left\{A_{p_{i,1}}^{T}e_{p_{i,1}}\right\} & \approx (-C_{1i}^{2}∥r_{i}∥\frac{\sigma_{\theta_{i}}^{2}}{2}\cos^{2}\alpha_{2i}-C_{1i}∥r_{i}∥\frac{\sigma_{\theta_{i}}^{2}}{2}\cos\alpha_{2i}\cos\alpha_{1i}\\ \; & \;+3C_{1i}C_{3i}∥r_{i}∥\sigma_{p_{i,1}}^{2}\cos^{2}\alpha_{2i}+C_{3i}∥r_{i}∥\sigma_{p_{i,1}}^{2}\cos\alpha_{2i}\cos\alpha_{1i}\\ \; & \;+C_{1i}^{2}∥d_{i}∥\frac{\sigma_{\theta_{i}}^{2}}{2}\cos^{3}\alpha_{2i}+C_{1i}∥d_{i}∥\frac{\sigma_{\theta_{i}}^{2}}{2}\cos^{2}\alpha_{2i}\cos\alpha_{1i}\\ & \;-3C_{1i}C_{3i}∥d_{i}∥\sigma_{p_{i,1}}^{2}\cos^{3}\alpha_{2i}-C_{3i}∥d_{i}∥\sigma_{p_{i,1}}^{2}\cos^{2}\alpha_{2i}\cos\alpha_{1i}\\ \; & \;-C_{1i}^{2}∥d_{i}∥\sigma_{\theta_{i}}^{2}\sin^{2}\alpha_{2i}\cos\alpha_{2i}+C_{1i}∥r_{i}∥\frac{\sigma_{\theta_{1}}^{2}}{2}\cos\alpha_{2i}\cos\alpha_{1i}\\ \; & \;+∥r_{i}∥\frac{\sigma_{\theta_{1}}^{2}}{2}\cos^{2}\alpha_{1i}-C_{1i}∥p∥\frac{\sigma_{\theta_{1}}^{2}}{2}\cos\alpha_{2i}\cos^{2}\alpha_{1i}\\ \; & \;-∥p∥\frac{\sigma_{\theta_{1}}^{2}}{2}\cos^{3}\alpha_{1i}+∥p∥\sigma_{\theta_{1}}^{2}\sin^{2}\alpha_{1i}\cos\alpha_{1i}\\ \; & \;+C_{1i}^{2}∥r_{i}∥\sigma_{\theta_{i}}^{2}\sin^{2}\alpha_{2i}-∥r_{i}∥\sigma_{\theta_{1}}^{2}\sin^{2}\alpha_{1i})∥r_{i}∥r_{i}^{T} \end{array}$$

It is clear that $E\left\{A_{p_{i,1}}^{T}e_{p_{i,1}}\right\}$ cannot be guaranteed to be zero for all i=2,…,N. Thus, $E\left\{A_{p_{i,1}}^{T}e_{p_{i,1}}\right\}\neq 0$.

Based on (41) and (43), we conclude that

(44) $$E\left\{A^{T}e\right\}=\sum_{i=1}^{N}E\left\{A_{\theta_{i}}^{T}e_{\theta_{i}}\right\}+\sum_{i=2}^{N}E\left\{A_{p_{i,1}}^{T}e_{p_{i,1}}\right\}\neq 0$$

which means $\delta_{LS}\neq 0$ and the least-squares estimate (35b) is biased.

### 5.2. WLS Solution and Bias Analysis

The weighted least-squares estimate for p is obtained from [43]

(45a) $$\begin{array}{cc} {\hat{p}}_{\mathrm{WLS}} & =\underset{p\in R^{2}}{arg min}{\left(Ap-b\right)}^{T}W_{\mathrm{PLE}}^{-1}\left(Ap-b\right) \end{array}$$

(45b) $$\begin{array}{cc} \; & ={\left(A^{T}W_{\mathrm{PLE}}^{-1}A\right)}^{-1}A^{T}W_{\mathrm{PLE}}^{-1}b. \end{array}$$

where $W_{\mathrm{PLE}}$ is the weighting matrix that approximates the covariance of the noise vector e:

(46) $$W_{\mathrm{PLE}}=E\left\{ee^{T}\right\}=\left[\begin{array}{ccc} W_{11} & W_{12} & W_{13}\\ W_{12}^{T} & W_{22} & W_{23}\\ W_{13}^{T} & W_{23}^{T} & W_{33} \end{array}\right].$$

The entries of $W_{\mathrm{PLE}}$ are given by

(47a) $$\begin{array}{cc} W_{11} & =E\left\{e_{\theta_{1,k}}^{2}\right\}\approx {∥p∥}^{2}\sigma_{\theta_{1}}^{2}, \end{array}$$

(47b) $$\begin{array}{cc} W_{12} & =E\left\{e_{\theta_{1,k}}\left[e_{\theta_{2,k}},\ldots ,e_{\theta_{N,k}}\right]\right\}=0_{1\times (N-1)},\\ W_{13} & =E\{e_{\theta_{1}}\left[e_{p_{2,1}},\ldots ,e_{p_{N,1}}\right]\}\\ \; & \approx [∥r_{2}{∥}^{2}∥p∥\sin\alpha_{12}-∥r_{2}{∥∥p∥}^{2}\sin\alpha_{12}\cos\alpha_{12},\\ \; & \;\ldots ,∥r_{N}{∥}^{2}∥p∥\sin\alpha_{1N}-∥r_{N}{∥∥p∥}^{2}\sin\alpha_{1N}\cos\alpha_{1N}]\sigma_{\theta_{1}}^{2} \end{array}$$

(47c) $$\begin{array}{cc} W_{22} & =E\{{\left[e_{\theta_{2}},\ldots ,e_{\theta_{N}}\right]}^{T}\left[e_{\theta_{2}},\ldots ,e_{\theta_{N}}\right]\}\\ \; & \approx \mathrm{diag}(\ldots ,∥d_{i}{{∥}^{2}\sigma_{\theta_{i}}^{2},\ldots )}_{i=2,\ldots ,N}, \end{array}$$

(47d) $$\begin{array}{cc} W_{23} & =E\{{\left[e_{\theta_{2}},\ldots ,e_{\theta_{N}}\right]}^{T}\left[e_{p_{2,1}},\ldots ,e_{p_{N,1}}\right]\}\\ \; & \approx \mathrm{diag}(\ldots ,(C_{1i}∥r_{i}{∥}^{2}∥d_{i}∥\sin\alpha_{2i}\\ \; & \;-C_{1i}∥r_{i}∥∥d_{i}{∥}^{2}\sin\alpha_{2i}\cos\alpha_{2i})\sigma_{\theta_{i}}^{2},\ldots {)}_{i=2,\ldots ,N} \end{array}$$

(47e) $$\begin{array}{cc} W_{33} & =E\{{\left[e_{p_{2,1}},\ldots ,e_{p_{N,1}}\right]}^{T}\left[e_{p_{2,1}},\ldots ,e_{p_{N,1}}\right]\}\\ \; & ={\left[\beta_{2,1},\ldots ,\beta_{N,1}\right]}^{T}\left[\beta_{2,1},\ldots ,\beta_{N,1}\right]\sigma_{\theta_{1}}^{2}\\ \; & \;+\mathrm{diag}(\ldots ,(C_{1i}{∥r_{i}∥}^{2}\sin\alpha_{2i}\\ & \;-C_{1i}∥r_{i}∥∥d_{i}∥\sin\alpha_{2i}\cos\alpha_{2i}{)}^{2}\sigma_{\theta_{i}}^{2},\ldots {)}_{i=2,\ldots ,N} \end{array}$$

(47f) $$\begin{array}{cc} \; & \;+{\left[\eta_{2,1},\ldots ,\eta_{N,1}\right]}^{T}\left[\eta_{2,1},\ldots ,\eta_{N,1}\right]W_{\mathrm{DRSS}}. \end{array}$$

where

(48a) $$\beta_{2,i}=∥r_{i}{∥}^{2}\sin\alpha_{1i}-∥r_{i}∥∥p∥\sin\alpha_{1i}\cos\alpha_{1i},$$

(48b) $$\eta_{2,i}=C_{2i}∥r_{i}∥∥d_{i}∥\cos^{2}\alpha_{2i}-C_{2i}{∥r_{i}∥}^{2}\cos\alpha_{2i}.$$

Note that $d_{i}$, $r_{i}$, $\alpha_{1i}$, $\alpha_{2i}$, $C_{1i}$ and $C_{2i}$ require prior knowledge of the source location p, which is not available. We replace p with ${\hat{p}}_{\mathrm{LS}}$ to calculate those terms.

Substituting (32) into (45b), we have

(49) $${\hat{p}}_{\mathrm{WLS}}=p-{\left(A^{T}W_{\mathrm{PLE}}^{-1}A\right)}^{-1}A^{T}W_{\mathrm{PLE}}^{-1}e$$

whence the estimation bias is obtained as

(50) $$\delta_{\mathrm{WLS}}=E\left\{{\hat{p}}_{\mathrm{WLS}}\right\}-p=-E\left\{{\left(A^{T}W_{\mathrm{PLE}}^{-1}A\right)}^{-1}A^{T}W_{\mathrm{PLE}}^{-1}e\right\}.$$

Similar to (39), for large *N*, (50) can be approximated as

(51) $$\delta_{\mathrm{WLS}}\approx -E{\left\{\frac{A^{T}W_{\mathrm{PLE}}^{-1}A}{2N-1}\right\}}^{-1}E\left\{\frac{A^{T}W_{\mathrm{PLE}}^{-1}e}{2N-1}\right\}.$$

As A and e are correlated as shown in Section 5.1, $E\{A^{T}W_{\mathrm{PLE}}^{-1}e\}\neq 0$, which implies $\delta_{\mathrm{WLS}}\neq 0$ and the WLS estimate is biased.

### 5.3. WIV Solution

The bias in the LS and WLS estimates can be significantly reduced by employing the method of instrumental variables. A weighted instrumental variable (WIV) estimator is obtained by introducing an IV matrix G which is strongly correlated with the matrix A while being statistically independent of e. The WIV solution is given by [45]

(52) $${\hat{p}}_{\mathrm{WIV}}={\left(G^{T}W_{\mathrm{PLE}}^{-1}A\right)}^{-1}G^{T}W_{\mathrm{PLE}}^{-1}b.$$

The IV matrix G is selected such that $E\left\{\frac{G^{T}W_{\mathrm{PLE}}^{-1}A}{2N-1}\right\}$ is nonsingular and $E\left\{\frac{G^{T}W_{\mathrm{PLE}}^{-1}e}{2N-1}\right\}\to 0$ as N→∞ [46]. A practical IV matrix that meets these requirements can be constructed from an initial source location estimate, such as the LS or WLS estimate, as described below. This procedure is based on [47]. Consider the following row partitioning of the IV matrix G:

(53) $$G={\left[G_{\theta_{1}}^{T},\ldots ,G_{\theta_{N}}^{T},G_{p_{2,1}}^{T},\ldots ,G_{p_{N,1}}^{T}\right]}^{T},$$

where

(54a) $$\begin{array}{cc} G_{\theta_{i}} & =\left[\sin{\hat{\theta }}_{i},\cos{\hat{\theta }}_{i}\right],\;i=1,\ldots ,N \end{array}$$

(54b) $$\begin{array}{cc} G_{p_{j,1}} & =\left(10^{-\frac{{\hat{p}}_{j,1}}{10\gamma }}\cos{\hat{\alpha }}_{2j}+\cos{\hat{\alpha }}_{1j}\right)r_{j}^{T},\;j=2,\ldots ,N. \end{array}$$

Here the AOA, triangle angle and DRSS estimates are obtained from the initial source location estimate $\hat{p}={\left[\hat{x},\hat{y}\right]}^{T}$ as

(55a) $$\begin{array}{cc} {\hat{\theta }}_{i} & =\tan^{-1}\left(\hat{y}-y_{i},\hat{x}-x_{i}\right),\;{\hat{\theta }}_{i}\in \left(-\pi ,\pi \right] \end{array}$$

(55b) $$\begin{array}{cc} {\hat{\alpha }}_{1j} & ={\hat{\theta }}_{1}-\vartheta_{1j},\;-\pi <{\hat{\alpha }}_{1i}\leq \pi \end{array}$$

(55c) $$\begin{array}{cc} {\hat{\alpha }}_{2j} & =\pi -{\hat{\theta }}_{j}+\vartheta_{1j},\;-\pi <{\hat{\alpha }}_{2j}\leq \pi \end{array}$$

(55d) $$\begin{array}{cc} {\hat{p}}_{j,1} & =10\gamma \log_{10}\frac{∥\hat{p}∥}{∥\hat{p}-r_{j}∥}. \end{array}$$

The bias of the WIV estimate is given by

(56) $$\begin{array}{cc} \delta_{\mathrm{WIV}} & =E\{{\hat{p}}_{\mathrm{WIV}}\}-p\\ \; & =-E\{{\left(G^{T}W_{\mathrm{PLE}}^{-1}A\right)}^{-1}G^{T}W_{\mathrm{PLE}}^{-1}e\} \end{array}$$

which, for sufficiently large *N*, can be approximated as

(57) $$\delta_{\mathrm{WIV}}\approx -E{\left\{\frac{G^{T}W_{\mathrm{PLE}}^{-1}A}{2N-1}\right\}}^{-1}E\left\{\frac{G^{T}W_{\mathrm{PLE}}^{-1}e}{2N-1}\right\}$$

where

$$E\left\{\frac{G^{T}W_{\mathrm{PLE}}^{-1}e}{2N-1}\right\}\approx 0.$$

As a result, $\delta_{\mathrm{WIV}}\approx 0$ and, therefore, the WIV estimate is approximately unbiased.

### 5.4. SHM-WIV Solution

A selective hybrid measurement method is introduced here to keep the IV matrix G, constructed from an initial source location estimate, and the data matrix A strongly correlated as there is a high probability that G and A lose correlation when the measurement noise is large [18]. The principle of selective hybrid measurements is to decide which rows of G should remain identical to those of A based on a measure of difference between them.

Consider the difference between the first *N* rows of A and G corresponding to the AOA measurements:

(58) $$\begin{array}{cc} A_{\theta_{i}}-G_{\theta_{i}} & =\left[\begin{array}{cc} \sin{\tilde{\theta }}_{i}-\sin{\hat{\theta }}_{i}, & \cos{\tilde{\theta }}_{i}-\cos{\hat{\theta }}_{i} \end{array}\right]\\ \; & =2\sin\left(\frac{{\tilde{\theta }}_{i}-{\hat{\theta }}_{i}}{2}\right)\left[\begin{array}{cc} \cos\left(\frac{{\tilde{\theta }}_{i}+{\hat{\theta }}_{i}}{2}\right), & -\sin\left(\frac{{\tilde{\theta }}_{i}+{\hat{\theta }}_{i}}{2}\right) \end{array}\right]. \end{array}$$

The common factor $\sin\left(({\tilde{\theta }}_{i}-{\hat{\theta }}_{i})/2\right)$ suggests that it will be appropriate to use the angle difference $|{\tilde{\theta }}_{i}-{\hat{\theta }}_{i}|$ as a measure of row difference [18], which leads to the following criterion for using ${\hat{\theta }}_{i}$, instead of ${\tilde{\theta }}_{i}$, in the *i*th row of the IV matrix G:

(59) $$|{\tilde{\theta }}_{i}-{\hat{\theta }}_{i}|\leq \lambda_{1}.$$

The recommended range of values for the threshold is $5\sigma_{\theta_{i}}\leq \lambda_{1}\leq 20\sigma_{\theta_{i}}$, i=1,…,N. Following extensive simulation studies, we have concluded that selecting $\lambda_{1}$ in this range achieves the intended effect of making the IV matrix strongly correlated with the data matrix. In general, the larger the angle noise, the larger $\lambda_{1}$ should be.

The row difference between A and G for the DRSS measurements is

(60) $$\begin{array}{cc} A_{p_{i,1}}-G_{p_{i,1}}\; & =\left(10^{\frac{{\tilde{p}}_{i,1}}{-10\gamma }}\cos{\tilde{\alpha }}_{2i}+\cos{\tilde{\alpha }}_{1i}\right)r_{i}^{T}-\left(10^{\frac{{\hat{p}}_{i,1}}{-10\gamma }}\cos{\hat{\alpha }}_{2i}+\cos{\hat{\alpha }}_{1i}\right)r_{i}^{T}\\ \; & =(\left(10^{\frac{{\tilde{p}}_{i,1}}{-10\gamma }}-10^{\frac{{\hat{p}}_{i,1}}{-10\gamma }}\right)\left(\cos{\tilde{\alpha }}_{2i}-\cos{\hat{\alpha }}_{2i}\right)+\left(10^{\frac{{\tilde{p}}_{i,1}}{-10\gamma }}-10^{\frac{{\hat{p}}_{i,1}}{-10\gamma }}\right)\cos{\hat{\alpha }}_{2i}\\ \; & \;+10^{\frac{{\hat{p}}_{i,1}}{-10\gamma }}\left(\cos{\tilde{\alpha }}_{2i}-\cos{\hat{\alpha }}_{2i}\right)+\left(\cos{\tilde{\alpha }}_{1i}-\cos{\hat{\alpha }}_{1i}\right))r_{i}^{T} \end{array}$$

where the following terms determine the magnitude of difference

(61a) $$\begin{array}{c} \left|10^{\frac{{\tilde{p}}_{i,1}}{-10\gamma }}-10^{\frac{{\hat{p}}_{i,1}}{-10\gamma }}\right| \end{array}$$

(61b) $$\begin{array}{c} |\cos{\tilde{\alpha }}_{1i}-\cos{\hat{\alpha }}_{1i}|=|2\sin\left(\frac{{\tilde{\alpha }}_{1i}+{\hat{\alpha }}_{1i}}{2}\right)\sin\left(\frac{{\tilde{\theta }}_{i}-{\hat{\theta }}_{i}}{2}\right)| \end{array}$$

(61c) $$\begin{array}{c} |\cos{\tilde{\alpha }}_{2i}-\cos{\hat{\alpha }}_{2i}|=|2\sin\left(\frac{{\tilde{\alpha }}_{2i}+{\hat{\alpha }}_{2i}}{2}\right)\sin\left(\frac{{\tilde{\theta }}_{1}-{\hat{\theta }}_{1}}{2}\right)|. \end{array}$$

From (61a) we obtain the following criterion for using ${\hat{p}}_{i,1}$, instead of ${\tilde{p}}_{i,1}$, in G:

(62) $$\begin{array}{c} |{\tilde{p}}_{i,1}-{\hat{p}}_{i,1}|\leq \lambda_{2} \end{array}$$

where the recommended range for $\lambda_{2}$ is $5\sigma_{p_{i,1}}\leq \lambda_{2}\leq 20\sigma_{p_{i,1}}$ with $\sigma_{p_{i,1}}=\sqrt{\sigma_{p_{1}}^{2}+\sigma_{p_{i}}^{2}}$. This range was confirmed to yield satisfactory estimation performance through extensive simulation studies.

Equations (61b) and (61c) result in the same criterion as (59). Thus, applying the difference measures in (59) and (62) to (60) yields the hybrid measurement selection criterion:

(63) $$|{\tilde{p}}_{i,1}-{\hat{p}}_{i,1}\left|\right|{\tilde{\theta }}_{1}-{\hat{\theta }}_{1}\left|+\right|{\tilde{p}}_{i,1}-{\hat{p}}_{i,1}\left|+\right|{\tilde{\theta }}_{1}-{\hat{\theta }}_{1}\left|+\right|{\tilde{\theta }}_{i}-{\hat{\theta }}_{i}|\leq \lambda_{1}\lambda_{2}+\lambda_{2}+2\lambda_{1}.$$

We refer to the WIV estimate incorporating (59) and (63) in the construction of the IV matrix G as the *selective hybrid measurement WIV* (SHM-WIV) algorithm.

## 6. Simulation Results

### 6.1. Simulation Set-Up

The RMSE and bias performance of the MLE, LS, WLS, WIV and SHM-WIV algorithms is compared using Monte Carlo simulations. The simulated network topology has ten sensor nodes at fixed locations and a source, all contained within a 60 m × 60 m region. The path loss exponent is assumed to be γ=4. The range of AOA and DRSS measurement noise is indicated by a noise index given in Table 1. The average SNR values for AOA and DRSS measurements are also included. AOA and DRSS measurements have different SNR values because the AOA noise power is the variance of the additive thermal (Gaussian) noise and the DRSS measurements are corrupted by the shadowing log-normal noise. The AOA measurements are obtained from an antenna array with m=10 elements, using [48]

(64) $${\mathrm{SNR}}_{i}=\frac{6}{m^{3}\sigma_{\theta_{i}}^{2}},\;i=1,\ldots ,N$$

which assumes the Cramer–Rao lower bound is achieved. The DRSS SNR values are for a source with transmit power of 40 dBm (10 W). The MLE uses the iterative Gauss–Newton method with initialization obtained from the LS estimate. The SHM-WIV threshold values are given in Table 2.

### 6.2. Fixed Source Location

**Figure 3:**
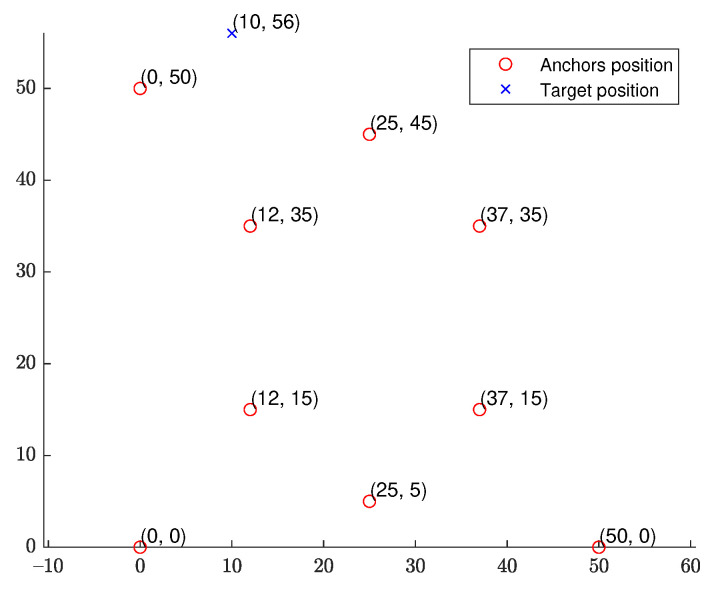
DRSS-AOA geometry with fixed source location.

**Figure 4:**
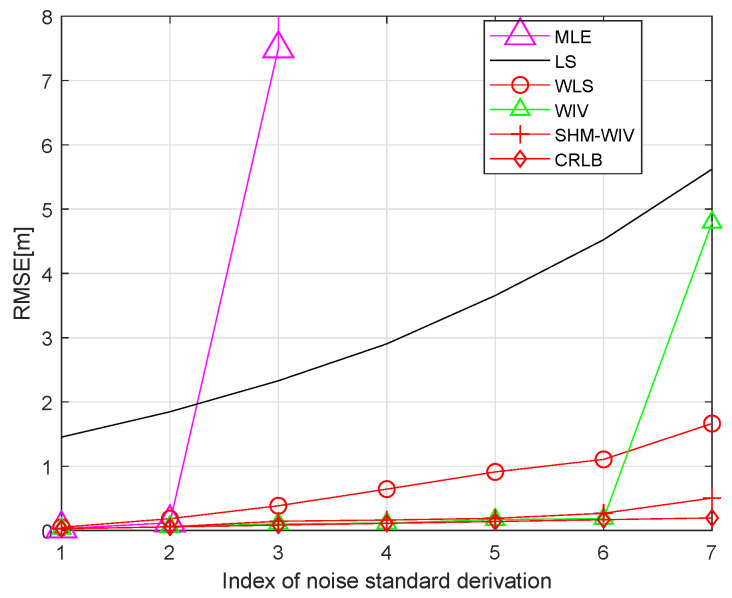
RMSE versus noise with fixed source location.

**Figure 5:**
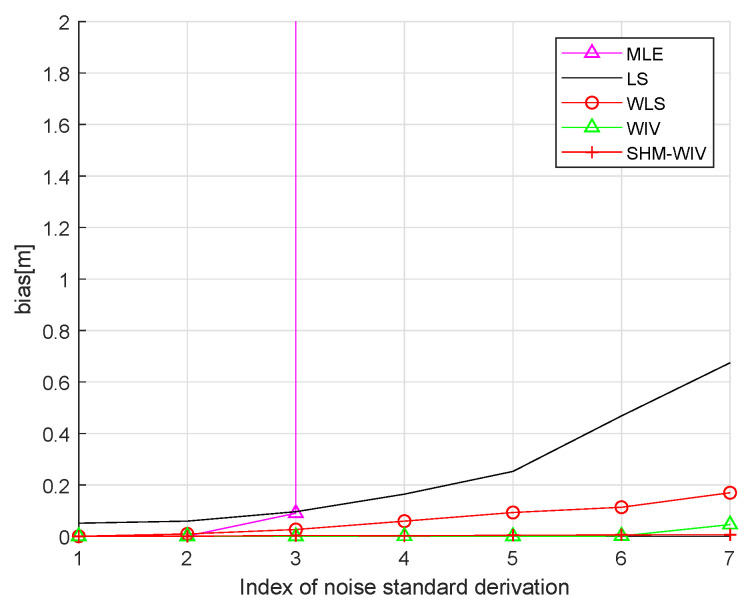
Bias versus noise with fixed source location.

We start with a fixed network topology simulation where the source is stationary at a fixed location $p={\left[10,56\right]}^{T}$ as shown in Figure 3. The simulations consist of 10,000 Monte Carlo runs. Figure 4 and Figure 5 present the RMSE and bias results versus noise index. The MLE achieves the CRLB at small noise (noise index 1 and 2), but starts to diverge for large noise. The LS exhibits significant bias and poor RMSE compared to the other estimates for all noise levels. The WLS has a better bias and RMSE performance than the LS, but still shows a large bias and deviates from the CRLB for large noise. The WIV attains the CRLB when the noise index is below 6, but its RMSE rapidly deviates from the CRLB at noise index 7. The SHM-WIV exhibits the best overall RMSE and bias performance for the entire noise range.

**Figure 6:**
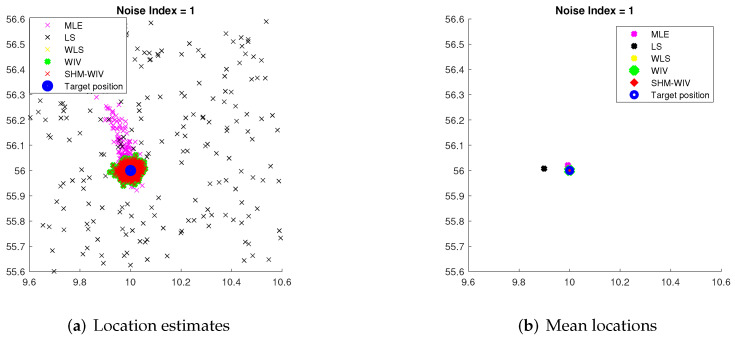
(**a**) Plot of individual location estimates for noise index 1; (**b**) Plot of mean location estimates for noise index 1.

**Figure 7:**
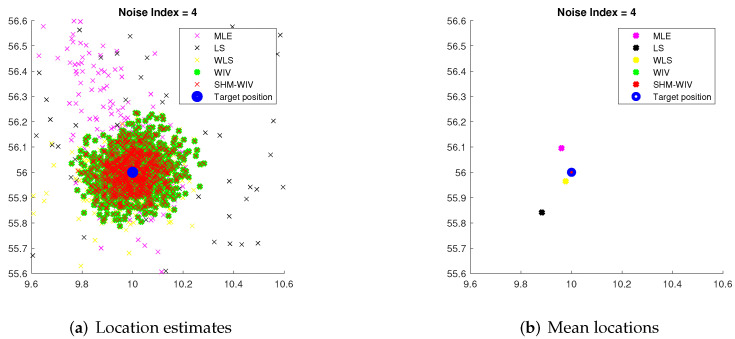
(**a**) Plot of individual location estimates for noise index 4; (**b**) Plot of mean location estimates for noise index 4.

For noise index 1 and 4, individual location estimates along with mean locations for the simulated algorithms are shown in Figure 6 and Figure 7, respectively, to demonstrate the spread of estimates. The standard deviations of Monte Carlo simulation results that led to the bias and RMSE values plotted in Figure 4 and Figure 5 are listed in Table 3. The standard deviation is left blank for algorithms that exhibit divergence.

The total run times of the simulated algorithms are listed in Table 4. We observe that the LS runs the fastest; however, it has a poor performance. The WLS is approximately three times slower than the LS due to weighting matrix computation. The MLE and WLS have comparable run times, even though the Gauss–Newton iterations can take longer time depending on initialization. The WIV is roughly five times slower than the LS because of computational overheads associated with weighting matrix and IV matrix computations. The SHM-WIV is slightly slower than the WIV method because of the additional SHM step.

### 6.3. Randomized Source Location

**Figure 8:**
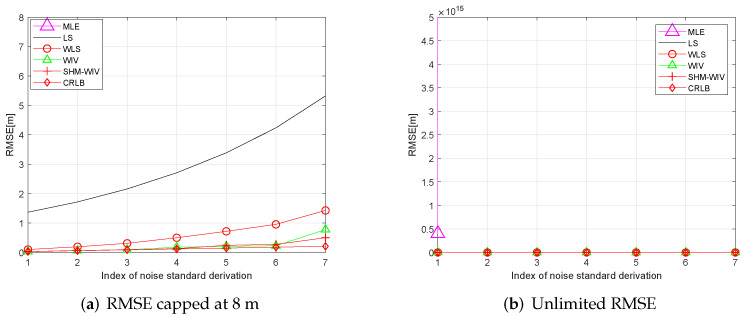
(**a**) RMSE versus noise with randomized source location (RMSE is capped at 8 m); (**b**) RMSE versus noise with randomized source location (MLE is missing in (**a**) as it diverges for entire noise range).

**Figure 9:**
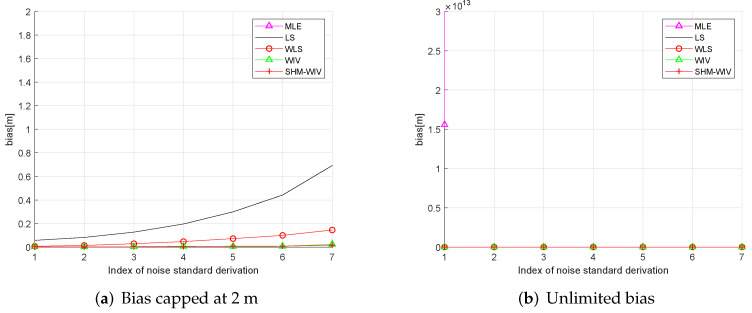
(**a**) Bias versus noise with randomized source location (bias is capped at 2 m); (**b**) Bias versus noise with randomized source location (MLE is missing in (**a**) as it diverges for entire noise range).

In these simulations 100 source locations are generated randomly in the 60 m × 60 m region, and, for each source location, RMSE and bias are evaluated using 10,000 Monte Carlo runs. The RMSE and bias results are shown in Figure 8 and Figure 9, respectively. The MLE has a divergence problem across the whole noise range. Among the remaining algorithms, the LS has the largest bias and RMSE. The WLS shows improved performance compared to the LS. The WIV and SHM-WIV have the best RMSE and bias performance with the SHM-WIV slightly outperforming the WIV at large noise levels.

## 7. Conclusions

A new pseudolinear hybrid DRSS-AOA localization method, free of nuisance parameter (squared source range from the reference sensor), was developed by exploiting the geometric relationship between AOA and DRSS measurements. To solve the resulting linear matrix equation for the source location, several variants of the pseudolinear estimator were proposed. These estimators are closed-form, leading to fewer computational steps than the MLE. However, the LS and WLS solutions have severe bias problems as verified by the simulations. The WIV estimator, on the other hand, was seen to be capable of alleviating the bias problem, achieving approximately zero bias for a large number of sensor measurements and small noise. The SHM-WIV was developed to guarantee a strong correlation between the IV matrix and the linearized data matrix for the WIV method as this correlation can be weakened when the noise is large. Simulation studies were carried out to compare the performance of the proposed estimators in fixed and randomized localization geometries. It was observed that the MLE has severe stability issues and cannot be considered an optimal solution at large noise. In the simulation studies the SHM-WIV outperformed all the estimators with an RMSE close to the CRLB and bias approaching zero.

## Figures and Tables

**Table 1:** Noise index for AOA/DRSS measurements.

Index	1	2	3	4	5	6	7
$\sigma_{\theta_{i}}$ (degrees)	0.1	0.2	0.3	0.4	0.5	0.6	0.7
$\sigma_{p_{i,1}}$ (dBm)	1	1.5	2	2.5	3	4	5
AOA SNR (dB)	32.95	26.92	23.40	20.90	18.96	17.38	16.04
DRSS SNR (dB)	−22.70	−23.20	−23.70	−24.20	−24.70	−25.70	−26.70

**Table 2:** $\lambda_{1}$ and $\lambda_{2}$ for SHM-WIV.

Noise Index	1	2	3	4	5	6	7
AOA $\lambda_{1}$	6.5σ	6.5σ	6.5σ	6.5σ	6.5σ	18σ	20σ
DRSS $\lambda_{2}$	6.5σ	6.5σ	6.5σ	6.5σ	6.5σ	18σ	20σ

**Table 3:** Standard deviations of Monte Carlo results for bias/RMSE values.

Index	bias/RMSE	MLE	LS	WLS	WIV	SHM-WIV
1	Bias	0.0007	0.0069	0.0004	0.0002	0.0001
	RMSE	0.0024	0.0098	0.0183	0.0147	0.0044
2	Bias	0.0013	0.0095	0.0013	0.0002	0.0003
	RMSE	0.0037	0.0115	0.0268	0.0019	0.0110
3	Bias	0.0017	0.0113	0.0029	0.0012	0.0008
	RMSE	0.0053	0.0123	0.0351	0.1219	0.0203
4	Bias	0.0033	0.0170	0.0045	0.0018	0.0012
	RMSE	0.1676	0.0159	0.0417	0.1596	0.0237
5	Bias		0.0184	0.0062	0.3103	0.0020
	RMSE		0.0206	0.0503	0.4246	0.0453
6	Bias		0.0233	0.0070	0.0044	0.0041
	RMSE		0.0294	0.0641	0.4337	0.4022
7	Bias		0.0288	0.0125	56.8201	0.0048
	RMSE		0.0302	0.0771	5682	0.4105

**Table 4:** Total simulation run time in MATLAB.

	MLE	LS	WLS	WIV	SHM-WIV
Time (s)	15.7860	5.5127	14.5313	24.3188	25.7447

## Data Availability

No new data were created or analyzed in this study. Data sharing is not applicable to this article.

## Appendix A. Derivation of Linearized DRSS Equation Noise

From (29), we have

(A1) $$\begin{array}{cc} e_{p_{i,1}} & =A_{p_{i,1}}p-b_{p_{i,1}}\\ \; & =\left(10^{\frac{{\tilde{p}}_{i,1}}{-10\gamma }}\cos\left(\alpha_{2i}-n_{i}\right)+\cos\left(\alpha_{1i}+n_{1}\right)\right)r_{i}^{T}p-{∥r_{i}∥}^{2}\cos\left(\alpha_{1i}+n_{1}\right). \end{array}$$

Expanding (A1), we obtain

(A2) $$\begin{array}{cc} e_{p_{i,1}} & =10^{\frac{{\tilde{p}}_{i,1}}{-10\gamma }}{∥r_{i}∥}^{2}\cos\alpha_{2i}\cos n_{i}+\sin\alpha_{2i}\sin n_{i}\\ \; & \;-10^{\frac{{\tilde{p}}_{i,1}}{-10\gamma }}∥r_{i}∥∥d_{i}∥\left(\cos^{2}\alpha_{2i}\cos n_{i}+\sin\alpha_{2i}\cos\alpha_{2i}\sin n_{i}\right)\\ & \;+∥r_{i}∥∥p∥\cos^{2}\alpha_{1i}\cos n_{1}-\sin\alpha_{1i}\cos\alpha_{1i}\sin n_{1}\\ \; & \;-∥r_{i}{∥}^{2}\cos\alpha_{1i}\cos n_{1}-\sin\alpha_{1i}\sin n_{1}. \end{array}$$

When the measurement noise is sufficiently small, we have

(A3a) $$\cos n_{i}\approx 1-\frac{n_{i}^{2}}{2},$$

(A3b) $$\sin n_{i}\approx n_{i},$$

and

(A3c) $$\begin{array}{cc} 10^{\frac{{\tilde{p}}_{i,1}}{-10\gamma }} & =10^{\frac{p_{i,1}}{-10\gamma }}10^{\frac{\epsilon_{i,1}}{-10\gamma }}\\ \; & \approx 10^{\frac{p_{i,1}}{-10\gamma }}\left(1-\frac{\epsilon_{i,1}}{10\gamma }\ln10+\frac{{\frac{\epsilon_{i,1}}{-10\gamma }\ln\left(10\right)}^{2}}{2}\right)\\ \; & \approx 10^{\frac{p_{i,1}}{-10\gamma }}-\frac{\epsilon_{i,1}}{10\gamma }10^{\frac{p_{i,1}}{-10\gamma }}\ln10+10^{\frac{p_{i,1}}{-10\gamma }}\frac{\epsilon_{i,1}^{2}\ln^{2}\left(10\right)}{200\gamma^{2}}. \end{array}$$

Substituting (A3) into (A2) yields

(A4) $$\begin{array}{cc} e_{p_{i,1}} & \approx -C_{1i}∥r_{i}{∥}^{2}\frac{n_{i}^{2}}{2}\cos\alpha_{2i}-C_{2i}{∥r_{i}∥}^{2}\epsilon_{i,1}\cos\alpha_{2i}\\ \; & \;+C_{2i}∥r_{i}{∥}^{2}\epsilon_{i,1}\frac{n_{i}^{2}}{2}\cos\alpha_{2i}+C_{3i}{∥r_{i}∥}^{2}\epsilon_{i,1}^{2}\cos\alpha_{2i}\\ \; & \;-C_{3i}∥r_{i}{∥}^{2}\epsilon_{i,1}^{2}\frac{n_{i}^{2}}{2}\cos\alpha_{2i}+C_{1i}{∥r_{i}∥}^{2}n_{i}\sin\alpha_{2i}\\ \; & \;-C_{2i}∥r_{i}{∥}^{2}\epsilon_{i,1}n_{i}\sin\alpha_{2i}+C_{3i}{∥r_{i}∥}^{2}\epsilon_{i,1}^{2}n_{i}\sin\alpha_{2i}\\ \; & \;+C_{1i}∥d_{i}∥∥r_{i}∥\frac{n_{i}^{2}}{2}\cos^{2}\alpha_{2i}+C_{2i}∥d_{i}∥∥r_{i}∥\epsilon_{i,1}\left(1-\frac{n_{i}^{2}}{2}\right)\cos^{2}\alpha_{2i}\\ \; & \;-C_{3i}∥d_{i}∥∥r_{i}∥\epsilon_{i,1}^{2}\left(1-\frac{n_{i}^{2}}{2}\right)\cos^{2}\alpha_{2i}-C_{1i}∥d_{i}∥∥r_{i}∥n_{i}\cos\alpha_{2i}\sin\alpha_{2i}\\ & \;+C_{2i}∥d_{i}∥∥r_{i}∥\epsilon_{i,1}n_{i}\cos\alpha_{2i}\sin\alpha_{2i}-C_{3i}∥d_{i}∥∥r_{i}∥\epsilon_{i,1}^{2}n_{i}\cos\alpha_{2i}\sin\alpha_{2i}\\ \; & \;+∥r_{i}{∥}^{2}\frac{n_{1}^{2}}{2}\cos\alpha_{1i}+∥r_{i}{∥}^{2}n_{1}\sin\alpha_{1i}-∥p∥∥r_{i}∥\frac{n_{1}^{2}}{2}\cos^{2}\alpha_{1i}\\ & \;-∥p∥∥r_{i}∥\cos\alpha_{1i}\sin\alpha_{1i}n_{1} \end{array}$$

where $C_{1i}=10^{-\frac{P_{i,1}}{10\gamma }}$, $C_{2i}=\frac{\ln10}{10\gamma }10^{-\frac{P_{i,1}}{10\gamma }}$ and $C_{3i}=\frac{\ln^{2}10}{200\gamma^{2}}10^{-\frac{P_{i,1}}{10\gamma }}$. Neglecting the second and higher-order noise terms in (A4), $e_{p_{i,1}}$ can be further simplified:

(A5) $$\begin{array}{cc} e_{p_{i,1}} & \approx -C_{2i}∥r_{i}{∥}^{2}\epsilon_{i,1}\cos\alpha_{2i}+C_{1i}{∥r_{i}∥}^{2}n_{i}\sin\alpha_{2i}\\ \; & \;+C_{2i}∥r_{i}∥∥d_{i}∥\epsilon_{i,1}\cos^{2}\alpha_{2i}-C_{1i}∥r_{i}∥∥d_{i}∥n_{i}\sin\alpha_{2i}\cos\alpha_{2i}\\ & \;+∥r_{i}{∥}^{2}n_{1}\sin\alpha_{1i}-∥r_{i}∥∥p∥n_{1}\sin\alpha_{1i}\cos\alpha_{1i}. \end{array}$$
